# Phenotypic and ancestry-related assortative mating in autism

**DOI:** 10.1186/s13229-024-00605-5

**Published:** 2024-06-14

**Authors:** Jing Zhang, J. Dylan Weissenkampen, Rachel L. Kember, iPSYCH Consortium, David M. Hougaard, Jonas Bybjerg-Grauholm, Thomas Werge, Thomas D. Als, Anders Rosengren, Jakob Grove, Anders D. Børglum, Elise B. Robinson, Edward S. Brodkin, Laura Almasy, Maja Bucan, Ronnie Sebro

**Affiliations:** 1 Department of Genetics, University of Pennsylvania, Philadelphia, PA USA; 2 Stanley Center for Psychiatric Research, Broad Institute of MIT and Harvard, Cambridge, MA USA; 3 Center for Genomic Medicine, Massachusetts General Hospital, Boston, MA USA; 4 Department of Psychiatry, University of Pennsylvania, Philadelphia, PA USA; 5 Center for Genomics and Personalized Medicine, Aarhus University, Aarhus, Denmark; 6 Department of Biomedicine (Human Genetics) and iSEQ Center, Aarhus University, Aarhus, Denmark; 7 Bioinformatics Research Centre, Aarhus University, Aarhus, Denmark; 8 The Lundbeck Foundation Initiative for Integrative Psychiatric Research, iPSYCH, Aarhus, Denmark; 9 Department of Psychiatry, Massachusetts General Hospital, Boston, MA USA; 10 Department of Biomedical and Health Informatics, Children’s Hospital of Philadelphia, Philadelphia, PA USA; 11 Department of Radiology, Mayo Clinic, Jacksonville, FL USA

**Keywords:** Assortative mating, Autism, Intellectual disability, Genetic ancestry, Polygenic scores, Linkage disequilibrium

## Abstract

**Background:**

Positive assortative mating (AM) in several neuropsychiatric traits, including autism, has been noted. However, it is unknown whether the pattern of AM is different in phenotypically defined autism subgroups [e.g., autism with and without intellectually disability (ID)]. It is also unclear what proportion of the phenotypic AM can be explained by the genetic similarity between parents of children with an autism diagnosis, and the consequences of AM on the genetic structure of the population.

**Methods:**

To address these questions, we analyzed two family-based autism collections: the Simons Foundation Powering Autism Research for Knowledge (SPARK) (1575 families) and the Simons Simplex Collection (SSC) (2283 families).

**Results:**

We found a similar degree of phenotypic and ancestry-related AM in parents of children with an autism diagnosis regardless of the presence of ID. We did not find evidence of AM for autism based on autism polygenic scores (PGS) (at a threshold of |r|> 0.1). The adjustment of ancestry-related AM or autism PGS accounted for only 0.3–4% of the fractional change in the estimate of the phenotypic AM. The ancestry-related AM introduced higher long-range linkage disequilibrium (LD) between single nucleotide polymorphisms (SNPs) on different chromosomes that are highly ancestry-informative compared to SNPs that are less ancestry-informative (D^2^ on the order of 1 × 10^−5^).

**Limitations:**

We only analyzed participants of European ancestry, limiting the generalizability of our results to individuals of non-European ancestry. SPARK and SSC were both multicenter studies. Therefore, there could be ancestry-related AM in SPARK and SSC due to geographic stratification. The study participants from each site were unknown, so we were unable to evaluate for geographic stratification.

**Conclusions:**

This study showed similar patterns of AM in autism with and without ID, and demonstrated that the common genetic influences of autism are likely relevant to both autism groups. The adjustment of ancestry-related AM and autism PGS accounted for < 5% of the fractional change in the estimate of the phenotypic AM. Future studies are needed to evaluate if the small increase of long-range LD induced by ancestry-related AM has impact on the downstream analysis.

**Supplementary Information:**

The online version contains supplementary material available at 10.1186/s13229-024-00605-5.

## Background

Positive assortative mating (AM) occurs when the spouse choice is based on phenotypic similarity [1]. If the phenotype is heritable, the consequences of AM on the genetic structure of the population include increased homozygosity (intra-locus correlations) and long-range linkage disequilibrium (LD) (inter-locus correlations) between unlinked markers, even between those on different chromosomes [2–6]. Ultimately, AM could lead to increased genetic variance over time and could contribute to increased disease prevalence and severity [7–9].

AM for several neuropsychiatric traits have been reported [8–15], including autism, which is a group of heterogenous heritable neurodevelopmental diagnoses [16], with individuals with cognitive impairment (CI) or intellectual disability (ID) and individuals with average or above average Intelligence Quotient (IQ) [17]. Positive correlations of autistic traits assessed by Social Responsiveness Scale (SRS) [18] and Broad Autism Phenotype Questionnaire (BAPQ) [19] in spouse pairs [9, 14, 20, 21] have been reported as evidence of phenotypic AM in autism. Genetic evidence of ancestry-related AM (spouse choice based on similarities in genetic ancestry) has been reported in parents of children with an autism diagnosis by evaluating the spousal correlation of genetic principal components (PCs) [9, 14] in two family based autism collection: the Autism Genome Project and the Simons Simplex Collection (SSC). Autism polygenic score (PGS) which captures the common genetic influence of autism was not correlated between parents of children with an autism diagnosis from a prior study [22].

Despite several research studies on AM in autism, there are still questions that remain. First, prior evidence suggested autism with ID (w/ ID) and without ID (w/o ID) might have different genetic architecture: de novo rare variants are more frequently observed among autism w/ ID than autism w/o ID [23–25], while higher single nucleotide polymorphism (SNP) heritability was observed in autism w/o ID compared to autism w/ ID, suggesting a more prominent role of common inherited variants in autism w/o ID [26]. Since AM can have an impact on the genetic architecture of a population, evaluating the pattern of AM in autism w/ and w/o ID separately could contribute to a more comprehensive understanding of the genetic architecture of the two subgroups of autism. Second, it is unknown what proportion of the phenotypic similarity between parents of children with an autism diagnosis could be explained by parents’ genetic similarity. Lastly, if there is genetic evidence of AM in autism, it is important to investigate if there are consequences on the genetic structure of the population, specificity, if there are increased homozygosity (intra-locus correlations) and induced long-range LD (inter-locus correlations) between unlinked markers.

To address these questions, we utilized two family-based autism collections: Simons Foundation Powering Autism Research for Knowledge (SPARK) [27] and SSC [28]. While both cohorts include genotype data, there are quantitative measures of autistic traits available for parents in SSC, whereas only autism status and limited demographic variables for parents are included in SPARK. Within families of European ancestry, we assessed phenotypic AM by evaluating correlations of quantitative autistic traits measured using the adult version of SRS [18] and BAPQ [19] (available in SSC), as well as autism and intelligence PGS between parents of children with an autism diagnosis. Population structure and ancestry-related AM were assessed by the spousal correlation of the genetic principal components (PCs) from principal components analysis (PCA) with 1000 Genomes European subpopulations [29]. We compared the degree of AM between autism w/ and w/ ID families and examined the proportion of phenotypic AM that can be explained by parents’ genetic similarity in SSC. We did not observe spousal correlations of autism PGS, but we confirmed the genetic evidence of ancestry-related AM in SSC and SPARK. Therefore, we further evaluated if there are intra-locus and inter-locus correlations introduced by the ancestry-related AM. The analysis included genotype data of 6300 participants and 322,042 SNPs in SPARK, and 8712 participants and 486,963 SNPs in SSC (Fig. S1).

## Methods

We analyzed the genetic and phenotypic data for SPARK [27] and SSC [28], downloaded from the Simons Foundation Autism Research Initiative (SFARI) base. The analysis of SPARK and SSC data was reviewed and approved by institutional review board at the University of Pennsylvania (IRB protocol number: 825701). The iPSYCH study was approved by Danish Data Protection Agency and the Scientific Ethics Committee in Denmark. The study is part of a PhD dissertation [30].

### 1000 genomes project

We used unrelated participants from the 1000 Genomes Project as the reference ancestry populations for the principal component analysis (PCA), specifically, the Utah Residents with Northern and Western European Ancestry from the United States (CEU), Yoruba from Ibadan, Nigeria (YRI), Han Chinese from Beijing, China (CHB), Japanese from Tokyo, Japan (JPT), Toscani from Italia (TSI), Finnish from Finland (FIN), British from England and Scotland (GBR), and Iberians from Spain (IBS) [29]. We kept autosomal SNPs with call rate $\ge$ 95% and Hardy–Weinberg Equilibrium (HWE) $\ge$ 1 × 10^–5^ in each population. We used CEU, YRI, CHB and JPT as the reference population for the first PCA to identify individuals with European ancestry. CEU, TSI, FIN, GBR and IBS were used as the reference population for the second PCA to better delineate European ancestry. We kept SNPs with minor allele frequency (MAF) $\ge$ 1 × 10^–2^ in the reference populations (Fig. S1A).

### SPARK

#### Genotyping

SPARK is an autism research initiative recruiting autistic probands and their families in the United States [27]. Participants were recruited from 32 clinical sites in the United States (Table S1) and were asked to complete a detailed questionnaire. The SPARK 201909 release (202002 update) includes 27,072 participants genotyped using Illumina Global Screening Array (GSA) v1 design using the Genome Reference Consortium Human Build 38 (GRCh38 human genome build).

#### Genotyping quality control

We removed participants who withdrew from the study and participants with questionable phenotypes (including lower confidence in autism diagnosis and suspected confounders to autism diagnosis including medical complications; more in supplemental notes). We restricted our analysis to participants from families with both parents, the proband and at least one unaffected sibling. We removed participants with more than 5% missing genotypes, and related families (closer than 2nd degree) based on kinship coefficients estimated using Kinship-Based Inference for genome-wide association studies (GWAS) (KING) [31]. Autosomal bi-allelic SNPs with call rate $\ge$ 95% were used in the analysis [5]. We restricted the analysis to SNPs that were common to both 1000 Genomes and SPARK. We excluded SNPs in regions of extended LD [32, 33], SNPs with MAF < 0.01 or HWE < 1 × 10^−5^. SNPs with greater than 5% Mendelian error rate were removed (Fig. S1B). We ended up with 322,042 SNPs in the analysis.

### SSC

#### Genotyping

SSC is ascertained in a slightly different manner to SPARK. SSC is a collection of more than 2000 families who have only one autistic child in each family [28]. SSC families were recruited from 12 sites (Table S2). SSC participants were genotyped on one of three platforms: Illumina 1Mv1 (n = 1354), Illumina 1Mv3 (n = 4626), or Illumina Omni2.5 (n = 4240) on *Homo sapiens* genome assembly National Center for Biotechnology Information NCBI36. For SSC, all genotypes were mapped to GRCh38 using LiftOver [34].

#### Genotyping quality control

We kept the SNPs that were common to all three of the SSC genotyping platforms and combined the SSC datasets. Participants who withdrew from the study were excluded. Family relationships were evaluated using KING by estimating kinship coefficients for all pairwise relationships [31]. Genotype patterns were consistent with the stated family relationships in all SSC families and no relationships of 2nd degree or closer were detected across families. We restricted the analysis to autosomal bi-allelic SNPs with call rate $\ge$ 95% that were common to 1000 Genomes and SSC. We excluded SNPs in regions of extended LD [32, 33], SNPs with MAF < 0.01, SNPs with Mendelian error rate greater than 5%, and SNPs with HWE p-value < 1 × 10^−5^ (Fig. S1C). The total number of SNPs in the final analysis is 486,963.

#### Principal components analysis for ancestry estimation

First, the continental ancestry for each SPARK and SSC participant was estimated. To do this, we used unrelated CEU, YRI, CHB, and JPT participants from the 1000 Genomes data. The PCA was performed using PLINK [35]. We used the first and second PCs to identify participants of European ancestry and removed all non-European participants from the analyses. Participants were assumed to be of European ancestry if their average PC1, and their average PC2 values were each both closer to that of the CEU participants than to that of the YRI and CHB/JPT participants (Fig. S2). We ended up with 1863 quartets and 420 trios in SSC, and 1586 quartets in SPARK. In cases where a family in SPARK has more than one unaffected sibling (n = 37), we prioritize selecting the sibling who shares the same sex as the proband, if available, and who is closest in age to the proband. We further excluded 11 SPARK families in which one or both parents had an autism diagnosis.

Next, we used 1000 Genomes participants of European ancestry (CEU, TSI, FIN, GBR, and IBS) to perform a second round PCA to better characterize the European ancestry in SPARK and SSC (Fig. S3). PC loadings from this round of PCA were used in the rest of the analyses. The absolute eigenvalues of PC1 from this round of PCA was used to identify ancestry-informative SNPs (SNPs that loaded the most heavily on |PC1|) in the intra-locus and inter-locus correlations analyses.

#### Autism and intelligence polygenic scores

We used SNP effect sizes and standard errors estimated from an external autism GWAS with 19,870 autistic individuals and 39,078 controls from the Danish Integrative Psychiatric Research (iPSYCH) consortium [36] to calculate autism PGS in SPARK and SSC.

To calculate the intelligence PGS, we used a large scale intelligence GWAS summary statistics [37]. SNPs that passed genotyping quality control and were common to the autism or intelligence GWAS summary statistics, 1000 Genomes, and the target dataset (SPARK or SSC) were used in the analysis.

PGS were calculated using LDpred2 [38]. LDpred2 adjusts the effect sizes from GWAS summary statistics by conditioning on a genetic architecture prior (the heritability explained by the genotypes and the fraction of causal markers) and LD information from a reference panel. We used the parents in SPARK or SSC for the LD references. We ran LDpred2 genome-wide using the ‘auto’ option to let LDpred2 automatically estimate the sparsity, p, and the SNP heritability, h^2^, from the summary statistics. The correlation between SNPs were calculated in a window size of 3 cM. For autism (a binary trait) PGS, we use SD_ss_ denote the standard deviations derived from the summary statistics, which for a binary trait, SD_ss_ = $\frac{2}{{se\left( {\widehat{{\gamma_{j} }}} \right)\sqrt {n_{eff} } }}$, where $n_{eff} = \frac{4}{{{1 \mathord{\left/ {\vphantom {1 {n_{case} }}} \right. \kern-0pt} {n_{case} }} + {1 \mathord{\left/ {\vphantom {1 {n_{control} }}} \right. \kern-0pt} {n_{control} }}}}$, $se\left( {\widehat{{\gamma_{j} }}} \right)$ is the standard error of the effect of variant j. SD_test_ denote the standard deviations of genotypes of participants in the study population (SD_test_ = $\sqrt {2*AF_{test} *\left( {1 - AF_{test} } \right)}$ where AF_test_ is the minor allele frequency of founders in the target population). As recommended by the authors of LDpred2, SNPs with SD_ss_ < 0.5·SD_test_ or SD_ss_ > 0.1 + SD_test_ or SD_ss_ < 0.1 or SD_test_ < 0.05 were removed (n_SPARK_ = 60, n_SSC_ = 14) [38]. Missing genotypes (< 5%) in SPARK and SSC were imputed with mean using *snp_fastImputeSimple()* function with “method = mean2” option in the *bigsnpr* [39] R package [40]. There were a total of 300,201 SNPs in SPARK and 475,058 SNPs in SSC included in the autism PGS calculation. There were a total of 307,058 SNPs in SPARK and 481,349 SNPs in SSC included in the intelligence PGS calculation. We used the polygenic transmission disequilibrium test (pTDT) [22] to evaluate if the polygenic influence of autism is over transmitted to autistic probands.

#### Cognitive impairment and intellectual disability in autistic probands

Probands in SPARK were assessed for cognitive impairment (CI), whereas probands in SSC were assessed for intellectual disability (ID). We divided families in SPARK and in SSC by whether the proband had cognitive impairment (in SPARK) or intellectual disability (in SSC) (autism w/ CI/ID) or not (autism w/o CI/ID) to evaluate if the degree of AM is different between these families. Criteria for likely cognitive impairment in SPARK were defined by nine variables related to the cognitive development of each proband (n_autism w/CI family_ = 707, n_autism w/o CI family_ = 867) (supplemental notes). In SSC, probands with full scale IQ < 70 were classified as having intellectual disability (n_autism w/ID family_ = 659, n_autism w/o ID family_ = 1618).

#### Correlations between spouses’ phenotypes, ancestry, and PGS

The Social Responsiveness Scale (SRS) adult version [18] obtained from an informant (mother reported on father and father reported on mother), and the self-reported Broad Autism Phenotype Questionnaire (BAPQ) [19] were available in SSC for parents. The results of these questionnaires were used as quantitative endo-phenotypes to better understand the genetic architecture of autism. The SRS adult version informant questionnaire measures core autistic traits on a continuous scale [18], and is made up of subscales which evaluate Awareness, Cognition, Mannerisms, Motivation, and Communication respectively [18]. The BAPQ self-report questionnaire measures the broader autism phenotype in three subscales: Aloof, Rigid, and Pragmatic Language [19]. The correlations between spouses’ measures of quantitative autistic traits in SPARK and SSC were evaluated using Spearman’s correlation coefficient [41]. The correlations between spouses’ genetic ancestry (the top two PCs from the PCA with 1000 Genomes participants of European ancestry), as well as autism and intelligence PGS (adjusted for age, sex, and the first 10 PCs from the PCA with 1000 Genomes participants of European ancestry) were evaluated using Pearson’s correlation coefficient. Spousal correlations in autism w/o CI/ID and autism w/ CI/ID families were compared using Fisher’s z-test with the *cocor* package [42] in *R* [40]. To adjust the significance level, we used the Bonferroni correction (divided the original Type I error rate, $\alpha$ (0.05) by the number of tests (n = 336, including subgroup analyses, see below)).

To investigate to what extent the spousal correlations of quantitative autistic traits could be explained by autism and intelligence PGS, genetic ancestry PCs, and demographic variables including sex, age, and highest education (predictors), we first built univariate regression models with parents’ SRS or BAPQ total scores as the dependent variable, and one of the predictors as the independent variable. Then, we built a full model with all predictors as independent variables. We reported the adjusted R-squared as a measure of the proportion of variance in parents’ SRS or BAPQ total scores that was explained by the independent variable(s). We then took the residuals of SRS and BAPQ total scores from each model (with sex variable removed) and recalculated the parents’ correlations of the residuals.

#### Carriers of rare variants with large effect

Rare de novo protein truncating variants (PTVs) and copy number variants in SPARK and SSC probands included in this paper have been analyzed and reported previously [43]. We identified all probands with a de novo PTV or copy number deletion in 373 neurodevelopmental disorder (NDD) related genes reported in Fu et al. [43] as carriers (n_carrier-SPARK_ = 74, n_carrier-SSC_ = 88).

#### Subgroup analyses

SSC is a simplex collection while SPARK includes both simplex (n = 1008) and multiplex families (n = 157; there are 410 families with unknown family type). There is also evidence supporting different genetic architectures of autism in males and females [44], and among individuals with and without a rare variant of large effect [23]. Therefore, we decided to compare the pattern of AM of autism w/ and w/o CI/ID in subgroups defined by above variables. To ensure adequate statistical power, we require each of the subgroups has a sample size of at least 85 (80% power to detect a correlation of 0.3 [14], assuming a Type I error rate of 0.05, Tables S3, S4). After examining the sample size, we compared the pattern of AM of autism w/ and w/o CI/ID in the following subgroups: simplex families in SPARK, as well as simplex families with male probands, simplex families with female probands, simplex families with probands without a rare de novo PTV or deletion in NDD genes, and simplex families with male probands without a rare de novo PTV or deletion in NDD genes in both SPARK and SSC. We also compared the pattern of AM between families with female probands and families with male probands in both SPARK and SSC.

#### Intra-locus correlations in SPARK and SSC

We first pruned SNPs using PLINK [35] with window size of 500 kb to remove SNPs with r^2^ > 0.1 from the SPARK dataset (now 90,621 SNPs). Then we used *SNPRelate* [45] in *R* [40] to randomly select SNPs that are at least 500 kb apart. This step was repeated 1000 times to create 1000 datasets, and each dataset contained approximately 3700–3800 SNPs. From the SNPs selected in each iteration, we identified the top 200 SNPs that loaded the heaviest on |PC1| and the bottom 200 SNPs that loaded the least on |PC1|. These top 200 SNPs are the SNPs that were most ancestry-informative for detecting population substructure, whereas the bottom 200 SNPs for |PC1| were less ancestry-informative and were used to serve as “controls”. These “control” SNPs were not neutral SNPs but were less ancestry-informative.

These steps were repeated in SSC quartets (n_family_ = 1863). After pruning, there were 63,583 SNPs left in SSC. Each of the 1000 iterations of randomly selecting SNPs that were 500 kb apart ended up with each dataset containing approximately 3600–3700 SNPs.

We calculated the intra-locus correlation coefficient using Wright’s F statistic, for the more ancestrally informative (top 200) and less ancestrally informative (bottom 200) SNPs in SPARK and SSC respectively. Consider a single bi-allelic marker (SNP) with alleles A, and a. If the observed number of Aa heterozygotes is $n_{oAa}$ and the expected number of Aa heterozygotes assuming Hardy–Weinberg Equilibrium (HWE) is $n_{eAa}$, then Wright’s F is:

1 $$F = 1 - \frac{{n_{oAa} }}{{n_{eAa} }}$$

We compared the distribution of Wright’s F between more ancestrally informative (top 200) SNPs for |PC1| and less ancestrally informative (bottom 200) SNPs for |PC1| in fathers, mothers, and unaffected siblings separately. Standard deviation was computed based on the mean Wright’s F distribution of the 1000 iterations. Two sample t-test was used to compare the mean Wright’s F between more ancestrally informative SNPs and less ancestrally informative SNPs. If there is no intra-locus correlation, the distribution of the mean Wright’s F for the more ancestrally informative and less ancestrally informative SNPs should not be statistically significantly different from each other.

To evaluate intra-locus correlations with a larger set of SNPs, we repeated this analysis for top 1000 SNPs for |PC1| and bottom 1000 SNPs for |PC1|.

#### Inter-locus correlations in SPARK and SSC

Next, we calculated the inter-locus correlation coefficient, using a linkage disequilibrium (LD) parameters D^2^ between two markers on different chromosomes. Consider two bi-allelic markers, the first SNP with alleles A and a; and the second SNP with alleles B and b. If the observed proportion of AB haplotypes is $p_{AB}$, the observed proportion of the A allele is $p_{A}$, and the observed proportion of the B allele is $p_{B}$, then:

2 $$D^{2} = \left( {p_{AB} - p_{A} p_{B} } \right)^{2}$$

Haplotype frequencies were calculated using the Expectation–Maximization algorithm [46]. We calculated D^2^ between pairs of SNPs that are more ancestry-informative (top 200 SNPs for |PC1|) and between pairs of SNPs that are less ancestry-informative (bottom 200 SNPs for |PC1|) on different chromosomes in fathers, mothers, and unaffected siblings separately. D^2^ were calculated separately in fathers, mothers, and unaffected siblings because these values are sensitive to the sample size used [47]. Standard deviation was computed based on the mean D^2^ distributions of the 1000 iterations. Two sample t-test was used to compare the mean D^2^ for more ancestrally informative SNPs to less ancestrally informative SNPs. If there is no inter-locus correlation, the mean D^2^ values for the more ancestry-informative SNPs should not be different from those calculated for the less ancestry-informative SNPs.

To evaluate inter-locus correlations with a larger set of SNPs, we repeated this analysis for top 1000 SNPs for |PC1| and bottom 1000 SNPs for |PC1|.

#### Quantification of assortative mating on autism

To quantify assortative mating on autism, we estimated the correlation ($\theta$) between genetic predictors of autism from SNPs on odd chromosomes and even chromosomes [48]. Following the method developed in Yengo et al., in parents of SPARK and SSC, we first selected SNPs on odd and on even chromosomes. We then conducted PCA in PLINK [35] to get the top 20 PCs using LD pruned SNPs (r^2^ > 0.1, > 1 Mb apart) on odd ($PCO$) and on even ($PCE$) chromosomes. Autism PGS from SNPs on odd ($S_{o}$) and even ($S_{e}$) chromosomes were calculated with the iPSYCH autism GWAS [36] summary statistics with SNP effect sizes adjusted by LDpred2 [38]. We fit the following two regressions to test $\theta$:

$$\begin{aligned} S_{o} & = \theta S_{e} + PCE_{1} + \cdots + PCE_{20} \\ S_{e} & = \theta S_{o} + PCO_{1} + \cdots + PCO_{20} \\ \end{aligned}$$

## Results

### Ancestry-related AM in autism w/ and w/o CI/ID families

**Fig. 1:**
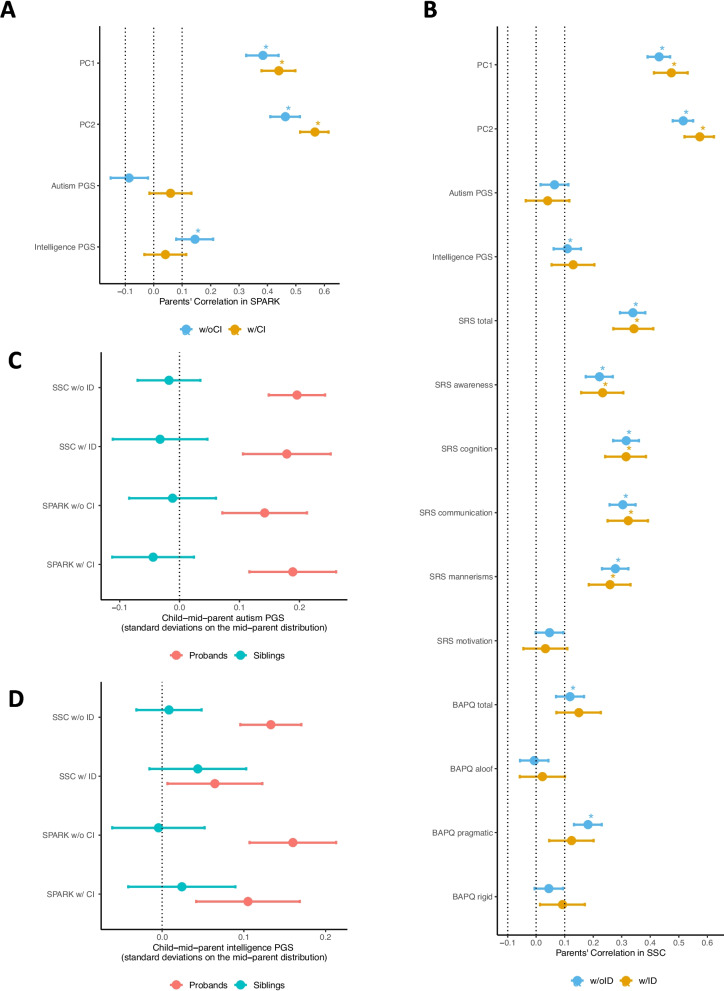
Phenotypic and ancestry-related AM in autism w/ and w/o CI/ID families of European ancestry in SPARK and SSC. **A** Spousal correlations and 95% confidence intervals of genetic ancestry PC1-PC2 (from the PCA with 1000 Genomes participants of European ancestry), autism PGS, and intelligence PGS in autism w/ and w/o CI families in SPARK. **B** Spousal correlations and 95% confidence intervals of genetic ancestry PC1-PC2 (from the PCA with 1000 Genomes participants of European ancestry), autism PGS, intelligence PGS, and measures of quantitative autistic traits (SRS and BAPQ) in autism w/ and w/o ID families in SSC. **C** Over-transmission of autism PGS and 95% confidence intervals in autism w/ and w/o CI/ID families in SPARK and SSC. **D** Over-transmission of intelligence PGS and 95% confidence intervals in autism w/ and w/o CI/ID families in SPARK and SSC. *p < 0.05/336 = 0.000149

To compare the pattern of ancestry-related AM in autism w/ and w/o CI/ID families in SPARK and SSC, we calculated the spousal correlations of PC scores from the second round of PCA (Supplemental results, Fig. S2CD) within SPARK (n_family_ = 1575) and SSC (n_family_ = 2283) participants of European ancestry along with 1000 Genomes European ancestry reference population (CEU, FIN, GBR, IBS, TSI). We observed similar degree of significant positive correlations between spouse-pairs’ PC1 scores (r_w/o CI_ = 0.38, r_w/ CI_ = 0.44, *P*_difference_ = 0.181) and PC2 scores (r_w/o CI_ = 0.46, r_w/ CI_ = 0.57, *P*_difference_ = 0.005) in SPARK w/ and w/o CI families (Fig. 1A, Table S5). There was also similar degree of significant positive correlations between spouse-pairs’ PC1 scores (r_w/o ID_ = 0.43, r_w/ ID_ = 0.47, *P*_difference_ = 0.241) and PC2 scores (r_w/o ID_ = 0.52, r_w/ ID_ = 0.57, *P*_difference_ = 0.074) in SSC w/ and w/o ID families (Fig. 1B, Table S12). After multiple testing corrections, no differences of spousal correlations between autism w/ and w/o CI/ID in SPARK and SSC remained statistically significant (Fig. 1AB, S3, S4, Tables S5, S12). We did not observe statistically significant differences in spousal correlations between autism w/ and w/o CI/ID families in the subgroup analyses after multiple testing corrections (Tables S6–S11, S13–S17).

### Spousal correlations of autism PGS in autism w/ and w/o CI/ID families

To evaluate whether the polygenic influence of autism is correlated in parents of autism w/ and w/o CI/ID probands, we calculated autism PGS within SPARK and SSC families of European ancestry using an external summary statistics from the iPSYCH consortium [36]. We found the polygenic influence of autism was over transmitted from parents to autistic probands in both w/ and w/o CI/ID families in SPARK and SSC (Fig. 1C, Table S18). When comparing autism PGS between autistic probands w/ CI/ID to autistic probands w/o CI/ID, we found no significant difference (beta_SPARK_ = 0.08, *P* = 0.12; beta_SSC_ = − 0.08, *P* = 0.09, Table S20). These results suggested the common genetic influence of autism is relevant in autism w/ and w/o CI/ID. We did not observe evidence of autism PGS based AM (at a threshold of |r|> 0.1) in either autism w/ or w/o CI/ID families in SPARK (r_w/o CI_ = − 0.09, r_w/ CI_ = 0.06, *P*_difference_ = 0.004) and SSC (r_w/o ID_ = 0.06, r_w/ID_ = 0.04, *P*_difference_ = 0.603) (Fig. 1AB, S3, S4, Tables S5, S12). There were no statistically significant differences in spousal correlations of autism PGS between autism w/ and w/o CI/ID families in all-sample analysis or subgroup analyses after multiple testing corrections (Fig. 1AB, Tables S5–S17).

### Spousal correlations of intelligence PGS in autism w/ and w/o CI/ID families

To better understand the polygenic influence on autism w/ and w/o CI/ID, we calculated intelligence PGS within SPARK and SSC families of European ancestry using a large scale intelligence GWAS summary statistics [37]. We found the polygenic influence of intelligence was over transmitted from parents to autistic probands in both w/ and w/o CI/ID families in SPARK and SSC, but the degree of over-transmission was lower in autism w/ ID probands (Fig. 1D, Table S19). We found significantly lower mean intelligence PGS in autistic probands w/ CI/ID compared to autistic probands w/o CI/ID (beta_SPARK_ = − 0.13, *P* = 0.01; beta_SSC_ = − 0.10, *P* = 0.01, Table S21). In parents, the intelligence PGS and autism PGS are not correlated (at a threshold of |r|> 0.01; r_SPARK_ = 0.03, *P* = 0.056; r_SSC_ = 0.05, *P* = 0.001). We observed weak spousal correlations of parents’ intelligence PGS in autism w/ and w/o CI/ID families in SPARK (r_w/o CI_ = 0.15, r_w/ CI_ = 0.04, *P*_difference_ = 0.039) and SSC (r_w/o ID_ = 0.11, r_w/ ID_ = 0.13, *P*_difference_ = 0.653). After multiple testing corrections, no differences of spousal correlations of intelligence PGS between autism w/ and w/o CI/ID in SPARK and SSC remained statistically significant in all-sample and subgroup analyses (Fig. 1AB, S3, S4, Tables S5–S17).

### Phenotypic AM in SSC

Phenotypic AM can be assessed using quantitative autistic traits assessed by SRS [18] and BAPQ [19]. We calculated the correlations of these traits between pairs of spouses of European ancestry in SSC. We observed moderate and significant positive spousal correlations (Fig. 1B, Table S12) of SRS total score (r_w/o ID_ = 0.34, r_w/ ID_ = 0.34, *P*_difference_ = 0.930), as well as awareness (r_w/o ID_ = 0.22, r_w/ ID_ = 0.23, *P*_difference_ = 0.804), cognition (r_w/o ID_ = 0.32, r_w/ ID_ = 0.32, *P*_difference_ = 0.992), communication (r_w/o ID_ = 0.30, r_w/ ID_ = 0.32, *P*_difference_ = 0.653), and mannerisms (r_w/o ID_ = 0.28, r_w/ ID_ = 0.26, *P*_difference_ = 0.666) subscales of SRS in both w/ and w/o ID families. We did not observe spousal correlation (at a threshold of |r|> 0.1) of SRS motivation subscale (r_w/o ID_ = 0.05, *P* = 0.061; r_w/ ID_ = 0.03, *P* = 0.410). The degree of spousal correlations of BAPQ total score and subscales were weaker (Fig. 1B, Table S12) than that of SRS but remained statistically significant in w/o ID families for the total score (r_w/o ID_ = 0.12, r_w/ ID_ = 0.15, *P*_difference_ = 0.511) and the pragmatic subscale (r_w/o ID_ = 0.18, r_w/ ID_ = 0.12, *P*_difference_ = 0.216). We did not observe spousal correlations (at a threshold of |r|> 0.1) for the aloof (r_w/o ID_ = − 0.01, *P* = 0.794; r_w/ ID_ = 0.02, *P* = 0.584) and the rigid (r_w/o ID_ = 0.04, *P* = 0.080, r_w/ ID_ = 0.09, *P* = 0.021) subscales. There were no significant differences in the degree of spousal correlations for the quantitative autistic traits between autism w/ and w/o ID families in all-sample and subgroup analyses. (Fig. 1B, Tables S12–S17). The spouse correlations for SRS and BAPQ total scores were slightly lower among parents of female probands compared to parents of male probands, but the results were not statistically significant (Table S15).

**Fig. 2:**
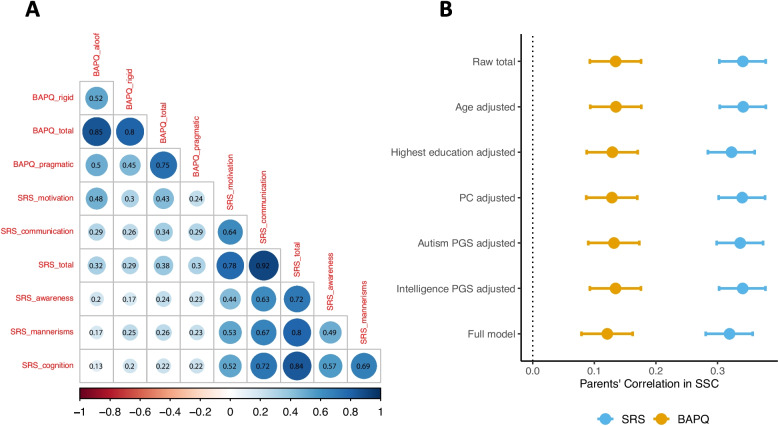
The proportion of phenotypic AM explained by ancestry-related AM, autism PGS, intelligence PGS, and demographic variables in SSC families of European ancestry. **A** Spearman’s correlation coefficients within and between SRS and BAPQ total scores and subscales for SSC parents. **B** The spousal correlations and 95% confidence intervals of SRS and BAPQ total scores after adjusting age, highest education, the top 10 genetic ancestry PCs, autism PGS, and intelligence PGS in SSC. Independent variables in the full model: age, highest education, top 10 genetic ancestry PCs, autism PGS, and intelligence PGS

In general, we observed higher spousal correlations for SRS total and subscale scores (except for Motivation subscale) than for BAPQ total and subscale scores (Fig. 2A). The pairwise correlation coefficients across SRS and BAPQ total and subscale scores ranged from 0.13 (between SRS cognition and BAPQ aloof) to 0.48 (between SRS motivation and BAPQ aloof) with most of the correlation coefficients on the order of 0.2–0.3, indicating low to moderate correlation between the two measures (Fig. 2A).

Since we observed similar AM patterns in autism w/ and w/o CI/ID families, we combined them for the rest of the analysis. Given the evidence of phenotypic AM observed in SSC, we evaluated whether the genetic ancestry PCs, the autism PGS, and the intelligence PGS could explain part of the spousal correlations in SRS and BAPQ. We first built univariate linear regression models to evaluate the proportion of variance of parent’s SRS or BAPQ total scores explained by genetic ancestry PCs, autism PGS, intelligence PGS, and a few demographic variables including sex, age, and highest education (Table 1). We found the top 10 genetic ancestry PCs explained 0.06% of the variance in SRS total scores and 0.5% of the variance in BAPQ total scores. The autism PGS explained 0.5% of the variance in SRS total scores and 0.1% of the variance in BAPQ total scores. The intelligence PGS explained 0.1% of the variance in SRS total scores and − 0.01% of the variance in BAPQ total scores (measured by adjusted R-squared). Genetic ancestry PCs, autism PGS, intelligence PGS, and demographic variables together explained 1.8% of the variance in parent’s SRS total scores and 8.1% of the variance in parent’s BAPQ total scores. The degree of the spousal correlations of SRS total scores (r = 0.341, *P* = 2.97 × 10^−62^) and BAPQ total scores (r = 0.135, *P* = 2.97 × 10^−10^) slightly reduced after adjusted for genetic ancestry PCs (r_SRS_ = 0.340, *P* = 7.07 × 10^−62^; r_BAPQ_ = 0.129, *P* = 1.77 × 10^−9^) and autism PGS (r_SRS_ = 0.337, *P* = 1.15 × 10^−60^; r_BAPQ_ = 0.132, *P* = 6.61 × 10^−10^). The spousal correlations of SRS total scores and BAPQ total scores reduced to 0.319 (*P* = 1.96 × 10^−54^) and 0.121 (*P* = 1.47 × 10^−8^) respectively after adjusting for PCs, autism PGS, intelligence PGS, and demographic variables (Fig. 2B, Table S22).

**Table 1:** The proportion of variance (adjusted R-squared) in SRS and BAPQ total scores explained by sex, age, highest education, the top 10 genetic ancestry PCs, autism PGS, and intelligence PGS in SSC

Model	BAPQ	SRS
Sex	0.068	− 0.0001
Age	0.003	− 0.00006
Highest education	0.009	0.012
Top 10 PCs	0.005	0.0006
Autism PGS	0.001	0.005
Intelligence PGS	− 0.0001	0.001
Full model	0.081	0.018

Independent variables in the full model: sex, age, highest education, top 10 genetic ancestry PCs, autism PGS, and intelligence PGS

### Intra-locus correlations

Since we observed genetic evidence of ancestry related AM (positive correlations between parents’ genetic ancestry PCs), we evaluated whether there are intra-locus correlations and inter-locus correlations induced by ancestry-related AM. For intra-locus correlations, we randomly selected SNPs that are at least 500 kb apart from a list of approximately independent SNPs (see Methods). This step was repeated 1000 times. From SNPs selected in each iteration, we identified the top 200 SNPs that loaded the heaviest on |PC1| as highly ancestry-informative SNPs and the bottom 200 SNPs that loaded the least on |PC1| as less ancestry-informative SNPs.

**Fig. 3:**
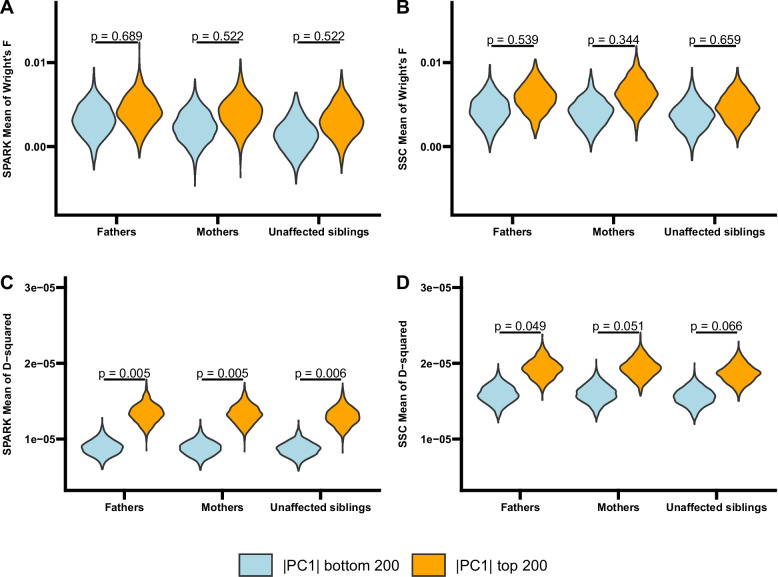
Intra-locus correlations (measured by Wright’s F) and inter-locus correlations (measured by D^2^) between SNPs on different chromosomes in SPARK and SSC families of European ancestry. **A** Mean Wright’s F at 200 SNPs that loaded the heaviest on |PC1| (|PC1| top 200) compared to mean Wright’s F at 200 SNPs that loaded the least on |PC1| (|PC1| bottom 200) in SPARK. **B** Mean Wright’s F at 200 SNPs that loaded the heaviest on |PC1| (|PC1| top 200) compared to mean Wright’s F at 200 SNPs that loaded the least on |PC1| (|PC1| bottom 200) in SSC. **C** Mean D^2^ between 200 SNPs that loaded the heaviest on |PC1| (|PC1| top 200) that were on different chromosomes compared to mean D^2^ between 200 SNPs that loaded the least on |PC1| (|PC1| bottom 200) that were on different chromosomes in SPARK. **D** Mean D^2^ between 200 SNPs that loaded the heaviest on |PC1| (|PC1| top 200) that were on different chromosomes compared to mean D^2^ between 200 SNPs that loaded the least on |PC1| (|PC1| bottom 200) that were on different chromosomes in SSC. **|PC1|:** the absolute value of the first PC from the PCA with 1000 Genomes participants of European ancestry

We compared Wright’s F (intra-locus correlation coefficient) at highly ancestry-informative SNPs to Wright’s F at less ancestry-informative SNPs. We observed a trend of increased homozygosity at highly ancestry informative SNPs compared to less ancestry informative SNPs in SPARK and in SSC, but this difference was not significant (Fig. 3AB, Table S23). This shows the fact that the HWE test has limited power to detect intra-locus correlations.

### Inter-locus correlations for SNPs on different chromosomes

To evaluate the inter-locus correlations induced by ancestry-related AM, we compared inter-locus correlation coefficient D^2^ between pairs of highly ancestry-informative SNPs that were on different chromosomes to D^2^ between pairs of less ancestry-informative SNPs that were on different chromosomes. The mean D^2^ value for highly ancestry-informative SNP pairs was larger in magnitude than the mean D^2^ for less ancestry-informative SNP pairs (Fig. 3CD, Table S23) in SPARK (fathers: *P* = 0.005; mothers: *P* = 0.005; unaffected siblings: *P* = 0.006) and in SSC (fathers: *P* = 0.049; mothers: *P* = 0.051; unaffected siblings: *P* = 0.066). However, the mean value of the induced D^2^ is small (on the order of 1 × 10^−5^). These results showed that there was increased LD introduced between the SNPs that were more strongly associated with population substructure, although the magnitude of the increase was small. The difference in the magnitude of the D^2^ values seen between SPARK and SSC may be attributable to the difference in the cohort sizes and the differences in the genotyping arrays used.

We observed a trend of decreased intra-locus and inter-locus correlations comparing parental generation to unaffected offspring in both cohorts (Fig. 3, Table S23), which shows progressive intermixing in the parental generation compared to the grandparental generation.

We repeated the analysis using the top and bottom 1000 SNPs based on the |PC1| loading and observed similar patterns (Fig. S5, Table S24).

### Quantification of assortative mating on autism

We quantified assortative mating on autism in SPARK and SSC by estimating the correlation between autism PGS from SNPs on odd and even chromosomes [48]. Under assortative mating, due to the induced inter-locus correlations, we expected the genetic predictor of a trait on odd chromosomes to be correlated with the genetic predictor of a trait on even chromosomes [48]. Applying this method to parents in SPARK and SSC, we did not observe a significant correlation between autism PGS from SNPs on odd and even chromosomes ($\theta_{SPARK} = - 0.0{2}0$, *P* = 0.274; $\theta_{SSC} = - 0.00{3}$, *P* = 0.836, Table S25).

## Discussion

In summary, we found autism w/ and w/o CI/ID in SPARK and SSC share a similar degree of positive ancestry-related AM within the participants of European ancestry. In SSC, using quantitative autistic traits measured by SRS and BAPQ, we found the degree of positive phenotypic AM was also similar in autism w/ and w/o ID. We did not observe evidence for autism PGS based AM (at a threshold of |r|> 0.1). The results hold when we stratified families by probands’ sex, and in subgroup analyses with only simplex families in SPARK or in probands without a de novo PTV or deletion in NDD genes. The adjustment of ancestry-related AM or autism PGS accounted for only 0.3–4% of the fractional change of the estimate of the phenotypic AM. The ancestry-related AM led to higher inter-locus correlations between SNPs on different chromosomes that are highly ancestry-informative compared to SNPs that are less ancestry-informative, although the mean value of the induced D^2^ is small (on the order of 1 × 10^−5^).

Despite the evidence of the potential different genetic architecture of autism w/ and w/o ID [23–26], we showed the pattern of phenotypic AM and ancestry-related AM (assessed using common variants) were similar in the two subgroups. We found the autism PGS was over transmitted to autistic probands w/ or w/o CI/ID in SPARK and SSC, indicating the common genetic influences of autism is likely relevant to both groups. Similar to a prior study [22], we did not find evidence of autism PGS based AM (at a threshold of |r|> 0.1). We further showed autism PGS only explained 0.5% of the variance in SRS and 0.1% of the variance in BAPQ. Our results showed the adjustment of spouses’ autism PGS, intelligence PGS, genetic ancestry PCs, age, and highest education reduced the positive spousal correlation of SRS and BAPQ total scores by 6.33% and by 10.01% respectively.

Our finding of positive spousal correlations for SRS and BAPQ scores in autism replicate results in prior studies [9, 14]. The magnitude of spousal correlations for BAPQ scores was lower compared to the spousal correlations for SRS scores (except for Motivation subscale). SRS measures the presence and severity of social impairment in autism [18]. However, BAPQ measures milder forms of autism symptoms outside of a definitive autism diagnosis [49]. SRS scores in SSC were based on informant-report (mother reports on father; father reports on mother), while BAPQ scores were self-reported. Lower spousal correlations for BAPQ compared to SRS are probably because the two questionnaires measure different domains and because of the difference of the informant.

The presence of AM is expected to alter the genetic architecture of heritable traits and can introduce biases in heritability estimates [48, 50, 51]. Given the genetic evidence of ancestry-related AM, we found induced inter-locus correlations between SNPs on different chromosomes that are highly ancestry-informative compared to SNPs that are less ancestry-informative. The spousal correlations of genetic ancestry PCs were moderate (on the order of 0.3–0.5), but the mean value of the induced inter-locus correlations measured by D^2^ were on the order of 1 × 10^−5^. Future studies are needed to evaluate whether this level of induced LD could have potential impact on the downstream analysis. This induced LD may not be fully controlled by adjusting for PCs since LD is a pair-wise phenomenon between two markers, while PCs only adjust for ancestry at a given locus.

## Limitations

SPARK and SSC were both multicenter studies. If the ancestries at each site were slightly different, and because most spouse-pair unions are geographically local, then there would be ancestry-related AM in SPARK and SSC due to geographic stratification. The study participants from each site were unknown, so we were unable to evaluate for geographic stratification. We only analyzed participants of European ancestry in SPARK and SSC, limiting the generalizability of our results to individuals of non-European ancestry [52, 53]. Another limitation is that we compared the top 200 SNPs for |PC1| to the bottom 200 SNPs for |PC1|. This was done to illustrate the effect of population substructure that could be seen in a genetically characterized relatively homogeneous European-American population. The degree of population substructure is likely higher in other non-European populations (African, Hispanic) and therefore the effects we describe will likely be lower in magnitude than that expected in several other non-European populations [54]. Our analysis only considered autosomal SNPs. No sex-linked variants were included. Finally, we did not examine if there was increased homozygosity or induced long-range LD for autism-associated SNPs compared to SNPs that are not associated with autism (e.g., top 200 SNPs with largest effect sizes in the autism GWAS summary statistics compared to the bottom 200 SNPs with no associations) due to a lack of spousal correlation of autism PGS. We could not rule out the possibility that the lack of spousal correlation of autism PGS is due to limited statistical power of PGS for autism.

## Conclusions

Within SPARK and SSC families of European ancestry, we found the patterns of phenotypic AM and ancestry-related AM (assessed using common variants) were similar in autism with and without CI or ID families. Common genetic influences of autism are likely relevant to both autism subgroups. Consistent with previous reports, we observed moderate spousal correlations of genetic ancestry (on the order of 0.3–0.5) and quantitative measures of autistic traits (on the order of 0.1–0.3). We did not observe spousal correlations of autism PGS (at the threshold of |r|> 0.1) among SPARK and SSC parents of children with an autism diagnosis. We further demonstrated that the adjustment of genetic ancestry and autism PGS accounted for < 5% of the fractional change of the spousal correlations of quantitative measures of autistic traits. We showed the spousal correlations of genetic ancestry (ancestry-related AM) led to higher long-range LD between genetic markers on different chromosomes that are highly ancestry-informative compared to genetic markers that are less ancestry-informative, although the mean value of the induced LD is small (D^2^ on the order of 1 × 10^−5^). Future studies are needed to evaluate if the small increase of long-range LD induced by ancestry-related AM has impact on the downstream analysis.

### Supplementary Information

Supplementary Material 1.

Supplementary Material 2.

## Data Availability

Data are available from https://base.sfari.org. Code can be provided upon request.

## Abbreviations

AM: Positive assortative mating
ID: Intellectually disability
SPARK: The Simons Foundation Powering Autism Research for Knowledge
SSC: The Simons Simplex Collection
PGS: Polygenic score
LD: Linkage disequilibrium
SNP: Single nucleotide polymorphism
CI: Cognitive impairment
IQ: Intelligence quotient
SRS: Social Responsiveness Scale
BAPQ: Broad Autism Phenotype Questionnaire
PC: Principal Component
Autism w/o ID: Autism without ID
Autism w/ ID: Autism with ID
PCA: Principal components analysis
SFARI: The Simons Foundation Autism Research Initiative
CEU: The Utah Residents with Northern and Western European Ancestry from the United States
YRI: Yoruba from Ibadan, Nigeria
CHB: Han Chinese from Beijing, China
JPT: Japanese from Tokyo, Japan
TSI: Toscani from Italia
FIN: Finnish from Finland
GBR: British from England and Scotland
IBS: Iberians from Spain
MAF: Minor Allele Frequency
GWAS: Genome-Wide Association Study
HWE: Hardy–Weinberg Equilibrium
GSA: Global Screening Array
iPSYCH: The Lundbeck Foundation Initiative for Integrative Psychiatric Research
pTDT: Polygenic transmission disequilibrium test
