# Supplementary material for: Phenotypic and ancestry-related assortative mating in autism
Source: Mol Autism. 2024 Jun 14;15:27. doi: 10.1186/s13229-024-00605-5 (PMC11177537; doi:10.1186/s13229-024-00605-5)
Supplement: Supplementary file 2 — Supplementary Material 2. [file 13229_2024_605_MOESM2_ESM.docx]

**Supplemental Materials**

**Supplemental notes:**

*Questionable phenotype and data inconsistencies in SPARK*

Individuals were removed if they have one of the following flags in the SPARK dataset:

asd_validity_flag: flag to alert to lower confidence in ASD diagnosis

asd_confound_flag: flag to alert to suspected confounds to ASD diagnosis including medical complications

age_validity_flag: flag to alert to suspected age errors and inconsistencies in age-related data entry

individual_data_validity: flag to alert to other data inconsistencies within individuals data

control_sibling_flag: flag to alert to potential developmental concerns in siblings which could affect sample inclusion

*Criteria for likely cognitive impairment in SPARK and intellectual disability in SSC.*

In SPARK, we defined probands with likely cognitive impairment if any of the following was true: 1) the proband had been diagnosed with intellectual disability or cognitive impairment; 2) if the proband was older than 4 years old at enrollment and did not use longer sentences of his/her own or was not able to tell the responder something that happened; 3) if the proband ever lost his/her speech and if the proband’s speech never came back to the level it was just before the loss and the proband did not use longer sentences of his/her own or was not able to tell the responder something that happened; 4) if the proband’s general cognitive ability for problem-solving and understanding concepts that do not require language was significantly below his/her actual age or grade level; 5) if the proband had cognitive (IQ or developmental) testing and had an overall IQ or developmental score below 70; 6) if the proband’s current level of spoken language was significantly below age; 7) for proband younger than 18 years old and with ratio IQ (midpoint of age level estimate for proband's current cognitive ability divided by age of proband at evaluation times 100) was below 70 (for such probands with age older than 12 years old, if their general cognitive ability for problem-solving and understanding concepts that do not require language were at age, they were classified as no cognitive impairment); 8) Vineland communication standard score was below 70 (if missing, used Vineland adaptive behavior composite standard score instead); 9) Full scale IQ test score was below 70 (if missing, used non-verbal IQ test score instead).

In SSC, probands with full scale IQ < 70 were classified as having intellectual disability.

**Supplemental results**

Principal component analysis and population substructure

To understand the population substructure in SPARK and SSC, we conducted PCA using unrelated Utah Residents with Northern and Western European Ancestry from the United States (CEU), Yoruba from Ibadan, Nigeria (YRI), Han Chinese from Beijing, China (CHB), and Japanese from Tokyo, Japan (JPT) from the 1000 Genomes reference populations to perform the first PCA (Figure S2) (1,2). The first PC separated participants with African ancestry from those without African ancestry in SPARK and SSC. The second PC separated participants with European ancestry from participants without European ancestry. We restricted the analysis to participants of European ancestry. Participants in SPARK and SSC were assumed to have European ancestry if their average PC1 and average PC2 values were each both closer to that of the CEU participants than that of the YRI and CHB/JPT participants from the 1000 Genomes (Figure S2AB). The final analysis was based on 1,575 families of European ancestry in SPARK and 2,283 families of European ancestry in SSC (Figure S1).

After restricting the analysis to participants of European ancestry, there remained substantial population substructure in both cohorts. We performed a second PCA on the SPARK and SSC participants along with 1000 Genomes participants of European ancestry (Figure S2CD): Toscani from Italia (TSI), Finnish from Finland (FIN), British from England and Scotland (GBR), Iberians from Spain (IBS), and CEU. The first PC better characterized European ancestry and showed clustering along a cline from North European ancestry to Southern European ancestry. The second PC showed clustering along a cline from North-Western European ancestry to Eastern/Southern European ancestry in both SPARK and SSC (Figure S2CD). The absolute value of the eigenvalues of PC1 was used to identify ancestry-informative SNPs (SNPs that loaded the heaviest on |PC1|).

| **Number** | **Site** | **City, State** |
| --- | --- | --- |
| 1 | Baylor College of Medicine, Texas Children's Hospital | Houston, TX |
| 2 | Boston Children's Hospital | Boston, MA |
| 3 | Center for Autism and the Developing Brain | White Plains, NY |
| 4 | Children's Hospital of Philadelphia | Philadelphia, PA |
| 5 | Children’s Specialized Hospital | New Brunswick, NJ |
| 6 | Cincinnati Children's Hospital Medical Center | Cincinnati, OH |
| 7 | Emory University and Marcus Autism Center | Atlanta, GA |
| 8 | Geisinger Autism & Developmental Medicine Institute | Lewisburg, PA |
| 9 | Kennedy Krieger Institute | Baltimore, MD |
| 10 | Maine Medical Center Research Institute | Scarborough, ME |
| 11 | Medical University of South Carolina | Charleston, SC |
| 12 | Nationwide Children's Hospital | Columbus, OH |
| 13 | Oregon Health and Science University | Portland, OR |
| 14 | The Rose F. Kennedy Children’s Evaluation & Rehabilitation Center at Montefiore | Bronx, NY |
| 15 | Rush University Medical Center | Chicago, IL |
| 16 | Stanford University | Stanford, CA |
| 17 | Stony Brook University | Stony Brook, NY |
| 18 | University of California, Davis MIND Institute | Sacramento, CA |
| 19 | University of California, Los Angeles | Los Angeles, CA |
| 20 | University of California, San Diego and Southwest Autism Research & Resource Center | La Jolla, CA and Phoenix, AZ |
| 21 | University of Colorado, Denver | Denver, CO |
| 22 | The University of Iowa | Iowa City, IA |
| 23 | University of Miami | Coral Gables, FL |
| 24 | University of Michigan | Ann Arbor, MI |
| 25 | University of Minnesota | Minneapolis, MN |
| 26 | University of Mississippi Medical Center | Jackson, MS |
| 27 | University of Missouri, Thompson Center for Autism and Neurodevelopmental Disorders** | Columbia, MO |
| 28 | The University of North Carolina at Chapel Hill** | Chapel Hill, NC |
| 29 | The University of Utah Child Development Program | Salt Lake City, UT |
| 30 | University of Washington** | Seattle, WA |
| 31 | Vanderbilt University Medical Center | Nashville, TN |
| 32 | Yale University | New Haven, CT |

**Table S1: SPARK clinical recruitment sites**

** Pilot sites

| **Number** | **Site** | **City, State** |
| --- | --- | --- |
| 1 | Baylor College of Medicine | Houston, TX |
| 2 | Children’s Hospital Boston/ Harvard Medical School | Boston, MA |
| 3 | Columbia University | New York, NY |
| 4 | Emory University | Atlanta, GA |
| 5 | McGill University | Montreal, QC |
| 6 | University of Michigan | Ann Arbor, MI |
| 7 | University of Illinois at Chicago | Chicago, IL |
| 8 | University of California, Los Angeles | Los Angeles, CA |
| 9 | University of Missouri | Columbia, MO |
| 10 | University of Washington | Seattle, WA |
| 11 | Vanderbilt University | Nashville, TN |
| 12 | Yale University | New Haven, CT |

**Table S2: SPARK clinical recruitment sites**

Table S3-S25 can be found in the supplemental excel sheets.

**Figure S1**: **Genotyping quality control and variant selection across all cohorts**

**(A)** genotyping quality control and variant selection across 1000 Genomes populations.

**(B)** genotyping quality control and variant selection in SPARK.

**(C)** genotyping quality control and variant selection in SSC.

**Figure S2**: **Principal component analysis of SPARK and SSC with 1000 Genomes**

**(A)** Principal component analysis of SPARK with 1000 Genomes YRI, CEU and CHB/JPT. SPARK participants (red dots) below the black line (the midpoint of the distance between CEU and YRI and the midpoint of the distance between CEU and CHB/JPT) were selected as participants of European ancestry.

**(B)** Principal component analysis of SSC with 1000 Genomes YRI, CEU and CHB/JPT. SSC participants (red dots) below the black line (the midpoint of the distance between CEU and YRI and the midpoint of the distance between CEU and CHB/JPT) were selected as participants of European ancestry.

**(C)** Principal component analysis of SPARK participants of European ancestry with 1000 Genomes CEU, FIN, GBR, IBS, and TSI.

**(D)** Principal component analysis of SSC participants of European ancestry with 1000 Genomes CEU, FIN, GBR, IBS, and TSI.

**Figure S3**: **Correlations of PC1 and PC2 loadings (from the principal component analysis with 1000 Genomes participants of European ancestry), autism PGS, and intelligence PGS between SPARK parents of autistic probands with cognitive impairment and SPARK parents of autistic probands without cognitive impairment**

**(A)** Correlations of PC1 and PC2 loadings, autism PGS, and intelligence PGS between SPARK parents of autistic probands with cognitive impairment.

**(B)** Correlations of PC1 and PC2 loadings, autism PGS, and intelligence PGS between SPARK parents of autistic probands without cognitive impairment.

**Figure S4**: **Correlations of PC1 and PC2 loadings (from the principal component analysis with 1000 Genomes participants of European ancestry), autism PGS, and intelligence PGS between SSC parents of autistic probands with intellectual disability and SSC parents of autistic probands without intellectual disability.**

**(A)** Correlations of PC1 and PC2 loadings, autism PGS, and intelligence PGS between SSC parents of autistic probands with intellectual disability.

**(B)** Correlations of PC1 and PC2 loadings, autism PGS, and intelligence PGS between SSC parents of autistic probands without intellectual disability.

**Figure S5**: **Intra-locus correlations (measured by Wright’s F) and inter-locus correlations (measured by D^2^) between SNPs on different chromosomes in SPARK and SSC families of European ancestry**

**(A)** Mean Wright’s F at 1000 SNPs that loaded the heaviest on |PC1| (|PC1| top 1000) compared to mean Wright’s F at 1000 SNPs that loaded the least on |PC1| (|PC1| bottom 1000) in SPARK.

**(B)** Mean Wright’s F at 1000 SNPs that loaded the heaviest on |PC1| (|PC1| top 1000) compared to mean Wright’s F at 1000 SNPs that loaded the least on |PC1| (|PC1| bottom 1000) in SSC.

**(C)** Mean D^2^ between 1000 SNPs that loaded the heaviest on |PC1| (|PC1| top 1000) that were on different chromosomes compared to mean D^2^ between 1000 SNPs that loaded the least on |PC1| (|PC1| bottom 1000) that were on different chromosomes in SPARK.

**(D)** Mean D^2^ between 1000 SNPs that loaded the heaviest on |PC1| (|PC1| top 1000) that were on different chromosomes compared to mean D^2^ between 1000 SNPs that loaded the least on |PC1| (|PC1| bottom 1000) that were on different chromosomes in SSC.

|PC1|: the absolute value of the first PC from the PCA with 1000 Genomes participants of European ancestry.

**Supplemental references**

1. 1000 Genomes Project Consortium. A global reference for human genetic variation. Nature. 2015 Oct 1;526(7571):68–74.

2. Purcell S, Neale B, Todd-Brown K, Thomas L, Ferreira MAR, Bender D, et al. PLINK: A Tool Set for Whole-Genome Association and Population-Based Linkage Analyses. American Journal of Human Genetics. 2007 Sep;81(3):559–75.
